# Peripheral leucocyte molecular indicators of inflammation and oxidative stress are altered in dairy cows with embryonic loss

**DOI:** 10.1038/s41598-021-91535-2

**Published:** 2021-06-17

**Authors:** Essa Dirandeh, M. A. Sayyar, Z. Ansari-Pirsaraei, H. Deldar, W. W. Thatcher

**Affiliations:** 1 Department of Animal Science, Sari Agricultural Sciences and Natural Resources University, P.O. BOX: 578, Sari, Mazandaran Iran; 2 Department of Animal Sciences, University of Florida, Gainesville, FL 32611 USA

**Keywords:** Animal physiology, Reproductive biology

## Abstract

Objective of experiment was to determine whether oxidative stress (OS) and inflammation altered embryonic loss in dairy cows. Blood samples were collected at days 0, 16, 32 and 60 after timed (AI) from 200 Holstein cows to determine embryonic loss based on interferon-stimulated gene-15 (ISG15) mRNA expression (day 16) and ultrasound at day 32 and day 60. Leucocyte expressions of mRNA *TLR2, TLR4, TNF-α, IL1B, IL10, STAT3* (inflammation), *PTGS2, PTGES* (prostaglandin synthesis), and *PLA2G4A* and *ALOX5AP* (eicosanoid metabolism) at days 0 and 16 were determined. Plasma redox status for antioxidant enzymatic activities of glutathione peroxidase (GPX), superoxide dismutase (SOD), total antioxidant capacity (TAC), and concentrations of malondialdehyde (MDA) were determined at days 0, 16, 32 and 60. All antioxidant-redox responses were beneficially significant in pregnant cows diagnosed pregnant at day16 and sustained pregnancy to day 60 compared to non-pregnant cows at day16 or pregnant at day16 and lost embryos by days 32 or 60. The leucocyte mRNA expressions of *TLR2, TLR4, STAT 3, IL1B, PTGS2, PLA2G4A* and *ALOX5AP* were greater and *PTGES* was lower at day16 in pregnant cows that lost embryos early (P < 0.05). In conclusion peripheral leucocyte molecular indicators of inflammation and plasma indicators of OS were altered in pregnant cows undergoing embryonic losses compared to cows with a sustained pregnancy.

## Introduction

Oxidative stress (OS) results from the imbalance between increased production of reactive oxygen and nitrogen species (free radicals) and the capacity of antioxidant mechanisms to neutralize these oxidants in tissues and blood. An imbalance (OS) can lead to reproductive diseases such as metritis, retained placenta and endometritis that contribute to decreased pregnancy rate, as well as mastitis that also is associated with reproductive disorders (Sordillo 2016)^1,2,3^. Pregnancy complications such as spontaneous abortion and recurrent pregnancy loss can also develop in response to OS^4^. Nazari et al.^2^ reported activities of glutathione peroxidase (GPX), superoxide dismutase (SOD) and total antioxidant capacity (TAC) postpartum were greater in multiparous Holstein dairy cows (n = 100), with normal luteal activity and lower in cows that lost their pregnancy compared with pregnant cows at days 32 and 60 after AI. Sayiner et al.^5^ reported in healthy cows that there is a relationship between blood antioxidant enzymes and metabolic parameters at different peripartum periods. High GPx and SOD activities, especially in the early period before and after parturition, is thought to be related to the adaptation of the animals to this process.

Leukocytes are recruited into tissues during inflammation through rolling and binding to endothelial cells followed by transmigration through the endothelium into tissue spaces. To initiate the inflammatory response, circulating leukocytes in the bloodstream must establish contact (tethering) with the vascular wall and adhere to it, while withstanding the shear forces. Tethering and rolling of the leukocytes over the activated endothelium are the first steps in the sequential process of extravasation^6^. Neutrophils (i.e., 25% of leukocytes in bovine peripheral blood of healthy animals) are the primary innate immune cells associated with clearing bacterial infections from the body^7^. Transcriptional studies confirmed the presence of bubaline endometrial Toll-like receptors (TLR) indicative that the uterus is equipped to mount TLR mediated responses following infection^8^. King et al.^9^ reported an increased expression of TLR 4 and 5 in third trimester of pregnancy may be related to defense mechanisms against infections. After bacterial challenge, resident cells recognize pathogen-associated molecular patterns, (PAMPs), via TLR, and initiate signaling events resulting in the production of proinflammatory cytokines and chemokines, and recruitment of inflammatory cells^10^. Because the sight of infection is continually exposed to pro-inflammatory cytokines and reactive oxygen metabolites (ROM), persistence of the inflammatory condition leads to chronic inflammation, OS, and consequently infertility^11^.

Pro-inflammatory and chemotactic cytokines play a key role in the recruitment and activation of phagocytic cells, which are main producers of Reactive Oxygen Species (ROS) and Reactive Nitrogen Species (RNS)^12^. Sina et al.^13^ reported inflamed cows had lower progesterone concentrations and smaller corpora lutea (CL) compared to healthy cows at first and second estrous cycles after calving. Inflammatory condition prolonged day to first service, decreased pregnancy/AI, and increased days open^13,14,15^. The specific mechanisms by which uterine infection and biochemical profiles disrupt ovarian function are many and diverse^16^. However, there is substantial evidence that the endotoxin lipopolysaccharide (LPS) is a key disruptor of ovarian function. LPS has been detected in follicular fluid of cattle with uterine disease^17^. Unsurprisingly, concentrations of LPS are directly correlated with bacterial load^14,15^.

It was hypothesized that OS, inflammatory conditions, and gene expression of leucocytes in postpartum lactating dairy cows are associated with reproductive responses after Timed AI (TAI). Furthermore, enzymatic and nonenzymatic antioxidant responses are activated and associated with fertility. Therefore, objectives were to determine whether pregnancy status at day 16 after TAI and subsequent embryo losses were associated with expression of leucocyte gene targets for inflammation, eicosanoids, and pregnancy; whether postpartum assessments of blood oxidant and antioxidants between 0 and 60 days after TAI were associated with pregnancy outcomes.

## Results

The percent of cows pregnant at day 16 following a TAI was 60% (120/200) based on diagnosis with ISG15 mRNA in blood cells (leucocytes). Percent of cows pregnant at the day 32 ultrasound diagnosis was 43% (86/200). Early embryo mortality between day 16 and day 32 was 28% (120-86/120) based upon ultrasound diagnosis at day 32 for presence of an intact conceptus with a heartbeat. Pregnancy per TAI at day 60 was 40% (80/200). A loss of six pregnancies between 32 and 60 days of pregnancy exemplifies a late embryo mortality of 7.0% (86–80/86).

### Sensitivity for pregnancy diagnoses at day 16 (ISG 15) and day 32 (ultrasound)

*ISG-15 sensitivity for pregnancy at d16* (correct pregnancy/correct pregnancy + incorrect pregnancy) was 71.6% sensitivity (86/86 + 34). Day 32 ultrasound was referent for pregnancy status assessment (“Supplementary Information”).

*Ultrasound sensitivity for pregnancy at ultrasound on d32* (correct pregnancy/correct pregnancy + incorrect pregnancy) was 93.0% sensitivity (80/80 + 6). Day 60 ultrasound was referent for pregnancy status.

### Specificity for pregnancy diagnoses at day 16 (ISG 15) and day 32 (ultrasound)

*ISG15 specificity for non-pregnancy at d16* (correct non-pregnancy/correct non-pregnancy + incorrect non-pregnancy) was 70.1% specificity (80/80 + 34). Day 32 ultrasound was referent for pregnancy status assessment.

*Ultrasound specificity for non-pregnancy at d32 ultrasound* (correct non-pregnancy/correct non-pregnancy + incorrect non-pregnancy) was 95.2% specificity (120/120 + 6). Day 60 ultrasound referent for pregnancy status.

### Relative mRNA expression of ISG15 on d 16 according to pregnancy status

**Figure 1:**
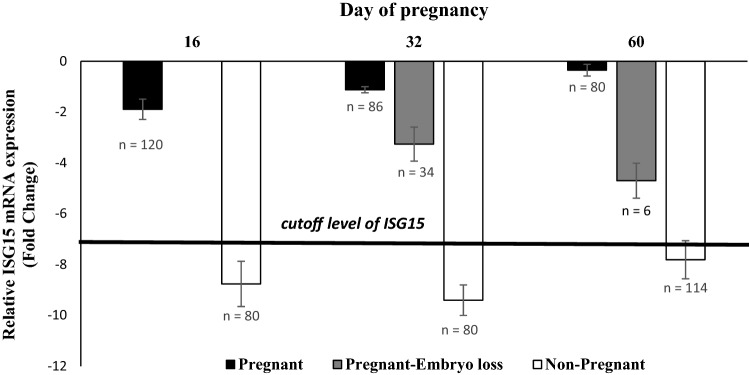
Relative mRNA expression of interferon stimulated gene ISG15 in peripheral blood leukocytes of samples on d 16 after AI, according to pregnancy statuses at 16, 32 and 60 days after AI. Error bars represent the 95% CI of the adjusted fold change. Black bars represent ISG15 expression at day 16 for cows diagnosed pregnant at days 16, 32 and 60 days. Shaded bars represent cows losing embryos between 16 and 32 days (n = 34) and 32–60 days (n = 6). The white bars on days 16 and 32 are ISG15 expression values for non-pregnant cows. The white bar on day 60 (non-pregnant): is an accumulative response of non-pregnant cows: 80 cows non-pregnant at day 16, 34 cows that lost embryos by day 32, and 6 cows losing embryos between days 32 and 60, which is a total of 114 non-pregnant cows.

Transcript expressions of ISG15 in PBL were 8.4- and 22.3-fold increases, respectively, in cows diagnosed pregnant compared with those diagnosed non-pregnant on days 32 and 64 after AI. Cows in pregnant-embryo loss group had intermediate values on day 32 after TAI (Fig. 1).

### Redox status

**Figure 2:**
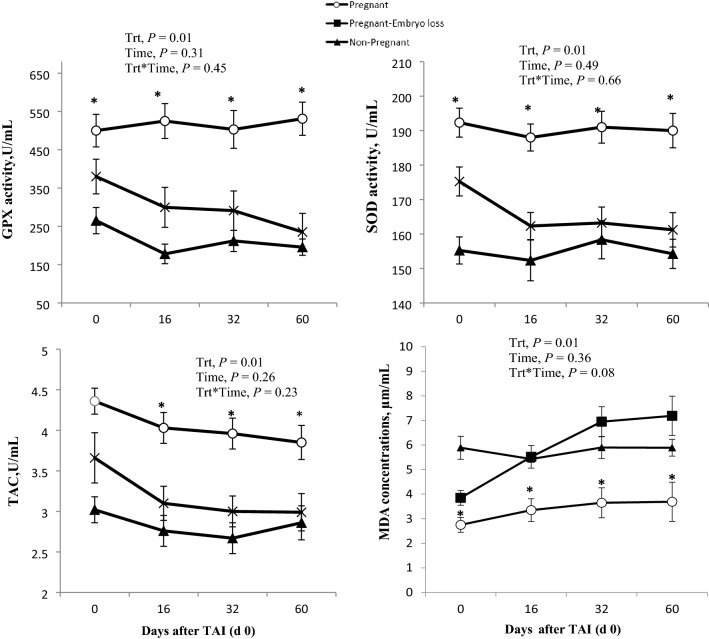
Mean activity of glutathione peroxidase (GPX, U/mL), superoxide dismutase (SOD, U/mL), total antioxidant capacity (TAC) and concentrations of malondialdehyde (MDA) of non-pregnant, pregnant and pregnant lactating dairy cows with embryo loss between day 16 and 32 days after TAI (day 0). Synchronization sequence started at 30 days postpartum. Values are Means ± standard error of means. Asterisks indicate a significant statistical difference at each specific day between pregnant (White) and non-pregnant (Black) lactating cows (P < 0.05).

The activities of GPX, SOD and TAC clearly were greater in lactating dairy cows that maintained pregnancy compared with those that experienced an embryonic loss between day 16 and day 32 or were non-pregnant to the TAI. (Fig. 2, P < 0.05). MDA concentrations were lower in pregnant cows compared with cows that had embryonic loss or were non-pregnant after TAI (Fig. 2, P < 0.05).

### Gene expression

#### Inflammation response

Experimental responses clearly documented increases in blood cellular (leucocyte) mRNA expressions of *TLR2, TLR4, STAT 3* and *IL1B* at both day 0 (time of TAI) and day16 in cows experiencing subsequent embryo loss by day 60 (n = 40) compared to cows that were pregnant (n = 80) (Table 1, P < 0.05). However, expressions of *TNF-α* and *IL10* mRNA did not differ significantly between pregnant cows and cows with embryonic loss (Table 1, P > 0.05).

**Table 1:** Effects of pregnancy status (pregnant or pregnant-embryo loss) on mRNA expression (log-2 scale) of genes related with inflammation response and eicosanoids in polymorphonuclear leukocytes at d 0 (TAI) and d16 after TAI.

Genes	Status of 16 day pregnant cows (n = 120)^a^							
	Pregnant (n = 80)		Pregnant-embryo loss (n = 40)		S.E.M	P-value		
	Day 0	Day16	Day 0	Day16		Status	Day	Interaction
**Inflammation**								
*STAT3*	1.23	1.59	3.12	4.01	0.29	0.01	0.59	0.68
*TLR2*	2.01	2.35	4.24	5.01	0.30	0.01	0.19	0.87
*TLR4*	1.89	2.96	3.61	5.89	0.36	0.04	0.33	0.52
*TNF-α*	0.68	0.71	0.92	0.97	0.12	0.76	0.25	0.60
*IL1B*	0.98	1.45	3.12	5.62	0.43	0.03	0.61	0.74
*IL10*	0.89	1.02	1.20	1.36	0.23	0.39	0.39	0.29
**Eicosanoids**								
*ALOX5AP*	2.20	2.66	5.25	6.11	0.37	0.01	0.46	0.39
*PLA2G4A*	2.18	2.89	6.12	6.85	0.48	0.01	0.67	0.51
*PTGS2*	2.59	2.89	5.82	6.66	0.59	0.01	0.59	0.78
*PTGES*	3.12	2.86	2.03	1.25	0.27	0.04	0.61	0.83

^a^Prediction of pregnancy status on d 16 after TAI was determined by ISG15 mRNA gene expression relative to b actin and at day 32 and day 60 after TAI as determined by transrectal ultrasonography. All cows were classified healthy. A cow was pregnant when diagnosed pregnant at 16, 32 and 60 days after TAI respectively. Cows were classified pregnant-embryo loss diagnosed pregnant at 16 days after TAI, but not pregnant at day 32 after TAI (n = 34) or pregnant at 32 days after TAI, but not pregnant at d 60 after TAI (n = 6).

#### Eicosanoid metabolism and prostaglandin synthesis

Increases were detected in blood cellular gene expressions of *PLA2G4A, ALOX5AP* and *PTGS2* mRNAs in pregnant cows with embryonic loss (n = 40) compared with pregnant (n = 80) cows at both days 0 and 16 (Table 2, P < 0.01). In contrast, *PTGES* mRNA expression was increased in pregnant cows compared to pregnant cows with embryonic loss (Table 2, P ≤ 0.05).

## Discussion

Interferon-τ (IFN-τ) produced profusely by the 16-day old conceptus induces endometrial synthesis and secretion of the ubiquitin cross-reactive protein now designated as ISG15^18^. Secretion of conceptus IFN-τ coupled with induction of endometrial ISG15 leads to an increase in gene expression of ISG15 mRNA in peripheral blood leucocytes (PBL) of pregnant cows (Gifford et al. 2007, Sinedino et al. 2017)^19,20,21^. Gene expression of ISG15 in PBL is a basis for diagnosis of non-pregnancy at day 18 after TAI^18^. The sensitivity (71.6%) and specificity (70.1%) of the pregnancy diagnosis at day 16 in the present study with 200 cows was approaching the sensitivity (81%) and specificity (75%) estimates at day 18^19^. Non-significant small increases in ISG-15 at day 16 were reported by^22^ (5 pregnant versus 15 non-pregnant cows) and^23^ (8 pregnant versus 21 non-pregnant cows). In the present study, a significant difference was detected in ISG 15 at day16 between cows subsequently classified as pregnant (n = 86) and non-pregnant (n = 114) by ultrasound diagnosis at day 32 (Fig. 1). The estimated pregnancy per AI of 60% at day 16, based upon a systemic diagnosis with PBL mRNA for ISG15 gene expression (Fig. 1), most likely reflects the intrauterine presence of viable embryos. Furthermore, intermediate ISG15 expression at day 16 may forecast pregnant cows that subsequently experience early or late embryonic mortality by 32 and 60 days after TAI (Fig. 1). These differences are indicative that day 16 conceptuses destined for loss likely had reduced capacity to secrete IFN-τ and therefore lower expression of ISG15 mRNA in PBL cells. Cows that sustained pregnancy to day 60 had the greatest PBL expression of ISG15 mRNA at day 16.

In addition to diagnosing non-pregnant cows at day 16, the possible partitioning of pregnant cows at day 16 that will sustain pregnancy from those that potentially lose pregnancy by day 32 offers potential for early re-synchronization strategies of non-pregnant cows. This would further advance the technological use of pregnancy diagnosis options for reproductive management^24^. Additional verification of early pregnancy diagnosis at day 16 or 18 and forecasting pregnancy losses, as part of a reproductive management program, warrants further investigation for further refinements of an ISG15 pregnancy test, and increases in number of experimental lactating cows. Development of sensitive systems for direct detection of IFN-τ may offer a novel sensitive alternative for reproductive management (Hansen et al. 2017)^25^.

Oxidative stress (OS) is a result of an imbalance between reactive oxygen species (ROS) and neutralizing capacity of antioxidant mechanisms^26,27^. Within the realm of this study, serum measurements of MDA represent a secondary product of lipid peroxidation as a marker of reactive oxygen species (ROS). Whole blood measurements of GPX, SOD and serum TAC were indicative of antioxidant responses. The peripheral clinical measurements to estimate OS differed significantly based upon reproductive status during the first 60 days after TAI.

A major focal response to evaluate ROS was measurement of MDA, which is a stable non-specific end-product of polyunsaturated fatty acid peroxidation. Fat metabolism during lactation is quite active due to the challenges of milk production, energy balance and supplementation of protected fatty acids as a nutraceutical^28,29^. Thus, MDA was a logical choice for the present experiment. Measures of antioxidants and oxidants were compatible from a clinical perspective of sampling, processing, and quantifying responses in many lactating cows over a long experimental period. Other more comprehensive methods to measure ROS, such as d-ROM need to be critically evaluated in future studies to improve understanding of relationships between oxidative stress and health. There is a possibility that relationships are not universal across markers representing various sources of oxidants, degree of OS, health status, and complexity of the experimental targets (e.g., general OS, differences of OS between cell types, degree of specificity involving HPLC, GC/MS, Spectrometry etc.).

Lactating cows that were pregnant had consistently higher plasma antioxidant responses compared to either non-pregnant or pregnant cows that experienced embryo mortality (Fig. 2). Peripheral diagnostic responses are indicative of pregnant status versus non-pregnancy and early embryonic losses. These changes may be associated with metabolic conditions of lactating cows but also associated with embryo health. Embryonic gene expression of Interferon-T, which stimulates endometrial secretion of ISG15 at day 16 also appears to stimulate gene PBL expressions of ISG-15 and candidate genes associated with both pregnancy and inflammation. Increased antioxidant defenses with a reduction in oxidant free radicals can create a more optimal OS due to a better balance between oxidants and antioxidants. Lower concentrations of MDA are indicative of less lipid peroxidation associated with lower lipid radicals and aldehydes in pregnant cows. These temporal targeted responses of OS appear to set the stage for potential reproductive success, since differences existed at the time of insemination, at approximately 60 DIM, and continued through 60 days after TAI. Nazari et al.^2^ first reported antioxidant status early in the postpartum period (21–40 days DIM) varied and was related to differences in categories of ovarian luteal activity (i.e., cows with normal luteal activity had greater antioxidant status than those with delayed first ovulation, short luteal phase, prolonged luteal phase, or anovulation). Nevertheless, following first TAI in a controlled synchronization program, day 60 pregnant cows had greater activities of the measured enzymatic antioxidants SOD, GPX, and nonenzymatic TAC (U/mL) and lower lipid peroxidation status at TAI, and days 32 and 60 than non-pregnant cows.

Expression of ISG15 in PBL for pregnancy diagnosis at day 16 and subsequent pregnancy losses was also the physiological foundation to examine collateral candidate genes of PBL that may be associated with both oxidation and pregnancy status of lactating cows. Specifically, PBL expression of a diversity of genes at days 0 and 16 after TAI in cows diagnosed pregnant at day16 versus pregnant cows that lost their embryos was examined. This represents a potential local dialogue between pregnancy status and gene expression of targeted genes associated with inflammation and eicosanoids in leucocytes at days 0 and 16 after TAI. The complement of target genes associated with inflammation (*STAT3*, *TLR2*, *TLR4, IL1B*; Table 2) was expressed greater in leucocytes collected on days 0 and 16 from cows that subsequently lost embryos by day 60 (n = 40), as compared to pregnant cows that maintained pregnancy by day 60 (n = 80). In contrast, leucocyte gene expressions of *TNF-α and IL10* at days 0 and 16 were not different between pregnant cows (n = 80) or pregnant cows that lost embryos (n = 40). Perhaps this is associated with differential expression of the various leucocytic cell types in blood. Damage induced by ROS can occur through modulation of cytokine expression and pro-inflammatory substrates via activation of redox-sensitive transcription factors AP-1 and p53^30^.

The increase in *IL1B* expression is relevant, since the pro-inflammatory cytokines (*IL1B and TNF-α*) activate the apoptotic cascade, causing cell death (Agarwal et al. 2012). The amplified gene expressions of leucocytes in pregnant cows losing embryos were inherent when cows were sampled at both TAI (Day 0) and Day 16. This may be emblematic of an inherent increase in the cow’s overall oxidation status contributing to a detrimental dialogue between developing embryo and oviductal/endometrial tissues. Overall enzymatic and nonenzymatic antioxidant responses in blood were low for a sustained period from days 0 to 60 days in non-pregnant or pregnant cows experiencing embryo loss.

Overall pregnancy loss from TAI to day 60 was 65%. Within the leucocyte series of blood cells, the two predominant cell types are neutrophils and lymphocytes. After pathogen detection, toll-like receptors (TLR2 and TLR4)^31^, activate signaling pathways (e.g., STAT3 and NF-κB), which result in production of pro-inflammatory cytokines^32^ such as *IL1B* in the present experiment. The *TLR2* is expressed most abundantly in blood leucocytes and mediates host response to Gram-positive bacteria. The *TLR4* gene is a member of the toll-like receptor family, which plays a fundamental role in pathogen recognition and activation of innate immunity. Macrophages that produce *IL1B* aid the predominate population of lymphocytes to fight infections reflecting an integration of other local cell types to produce inflammatory mediators. In contrast, *IL10* is a cytokine with potent anti-inflammatory properties and was not overly expressed supporting overall coordination of sustaining an inflammatory environment in cows that experienced embryonic loss. Prolonged inflammation and its carryover effects impair immune status and reproductive performance of dairy cows^11,33^. Up-regulation of genes associated with the pro-inflammatory cascade (*TLR2, TLR4* and *IL1B*) in peripheral blood of leucocytes in cows at 16 days after TAI was evident in cows with embryonic loss compared with pregnant cows. Pathogen elimination and tissue remodeling processes may be impaired in cows with embryonic loss.

Neutrophil stimulation produces ROS and lysosomal enzymes as well as pro-inflammatory and anti-inflammatory mediators, which include bioactive lipids such as eicosanoids: prostaglandins, leukotrienes and thromboxanes. These are lipid-based cellular hormones that regulate hemodynamics, inflammatory responses, and other intracellular pathways. Furthermore, the endocannabinoid system also regulates inflammation and is altered in the endometrium of cows experiencing early embryonic loss. Such cows had increased mRNA expressions of CNR1 receptor and synthetic NAPEPLD and decreased mRNA expression of the hydrolyzing enzyme (FAAH)^34^.

Results of present study also revealed increases in PBL mRNA expression of genes related to eicosanoid metabolism in healthy cows with embryonic loss compared with healthy pregnant cows at day 16. Increase PLA2G4A mRNA expression in cows with embryonic loss would contribute to an increase in the hydrolysis of cell membrane phospholipids to release arachidonic acid, which subsequently could be used for leukotriene synthesis since mRNA expression of ALOX5AP was increased.

The expression of PBL *PTGS2* mRNA was increased in pregnant cows with embryonic loss, but *PTGES* mRNA expression was increased in PBL of pregnant cows. Within the endometrium of day 15 cyclic cows, PGE_2_ secretion of cultured stromal cells (ng µg^−1 DNA^) is 12 times greater than luminal epithelial cells^35^. The dialogue between PBL and the underlying stromal and epithelial cell environment at day 16 of pregnancy is intriguing. Prostaglandins play a key role in the generation of an inflammatory response because of their pro-inflammatory properties and are responsible for typical signs of inflammation, luteal regression, and embryonic loss^36^. The anti-inflammatory response opposes the host inflammatory prostaglandin response, which potentially limits harmful collateral damage to tissues and predispose cells in the resolution of inflammation^37^. The localized balance between anti-inflammatory and inflammatory dialogue of PBL within the uterine environment at day 16 may be associated with immune-modulatory systems within the uterus associated with onset and maintenance of pregnancy. IFNT alters expression of immune function genes broadly during early pregnancy in ruminants, as reviewed by Ott^39^. Specifically bovine neutrophils treated with IFNT, ex vivo, increased expression of several ISGs, showing that this most abundant blood leukocyte does respond to IFNT. Specific cell types were not evaluated in the present study. Originally ISG15 was named bovine ubiquitin cross-reactive protein (boUCRP) because it could be detected on Western blots using a ubiquitin polyclonal antiserum (Austin et al. 2004).^38,18^

Inflammasomes are cytosolic multiprotein complexes that serve as platforms for recruitment and activation of Caspase-1 protease, which leads to processing and maturation of cytokines such as interleukin-1β activation leading to a form of cell death^40^. The ISG15, which appears to be an ubiquitin homolog, is not constitutively expressed in cells but induced by IFNT produced by the early bovine embryo. Addition of ubiquitin to cellular proteins appears to be a key to regulation of the innate immune response and possibly bovine ISG15 may exert a similar function with an array of targeted proteins in interferon-stimulated cells. In the present study with pregnant lactating cows, those that lost their embryos had PBMs with lower ISG15 expression, increased expressions of inflammatory genes including interleukin-1β, and differential expression of eicosanoids associated with inflammatory prostaglandin responses.

## Methods

Experiment was conducted on a modern commercial dairy farm (with 3500 milking cows) in northern Iran (Longitude E 53.06 and Latitude N 36.33) during summer (June–November 2018). All animal procedures were approved by the Iranian Ministry of Agriculture (Permission no. 2018.06.01).

### Cow management

All healthy multiparous cows (n = 200, milk yield of 35.0 ± 0.5 kg/day, body condition score (BCS) of 3.0 ± 0.25 and 3.1 ± 1.2 parity) were synchronized using a MG6GP timed-AI (TAI) protocol initiated at 28 + 3 days postpartum (Cows received PGF2α (500 μg CLOPROSTENOL, Parnell Technologies, Alexandria, Australia) 4 days later GnRH (100 μg GONADORELIN ACETATE, Parnell Technologies) followed 6 days later by an Ovsynch56 TAI program^41^. Pregnancy status was predicted via blood cell ISG15 mRNA gene expression at day 16 after TAI. Prediction of pregnancy at day16 was based on blood ISG15 mRNA level greater than − 7.0^42^. Pregnancy at days 32 and 60 after TAI were diagnosed via transrectal ultrasonography (7 mH probe,EASI-SCAN version 3, BCF Technology Ltd.. All cows were considered healthy (n = 200 and partitioned into non-pregnant at day 16 (n = 80, pregnant day 16 (n = 120, pregnant day 32 (n = 86, and cows experiencing embryonic losses between days 16–32 (n = 34 and days 32–60 (n = 6).

### Health status

All cows were monitored daily (8.00 a.m.) for signs of diseases. Only cows with natural calving and without dystocia, retained fetal membrane (RFM) or infectious diseases (metritis and endometritis) were utilized in this study. Cows were considered to have RFMs if fetal membranes were not expelled within the first 24 h postpartum^2^. All cows were checked once daily by the herd veterinarian for rectal temperature (fever was defined as a rectal temperature of > 39.5 °C) and for signs of metritis during the first 14 days of lactation (metritis was characterized by presence of watery, fetid vaginal discharge, and rectal temperatures > 39.5 °C within the first 14 days after calving^11^. Clinical endometritis was evaluated at 20 ± 1 and 40 ± 2 days in milk (mean ± SEM) by assessing vaginal mucus as described previously^11^. Cows having mucopurulent or purulent discharge were classified as having clinical endometritis. Based on above criteria 50 cows diagnosed sick, mastitis (n = 5), locomotor (n = 10), metabolic diseases (n = 25), infectious diseases (n = 10) and just 10 of them received antibiotics.

### Blood collection and analyses

Blood samples were collected from coccygeal vessels into heparinized evacuated tubes, placed in ice bath and taken immediately to the laboratory either on days 0 and 16 of pregnancy for gene expression (i.e., for measurements of *ISG15, TLR2, TLR4, TNF-α, STAT3, IL1B, IL10, PTGS2, PTGES, PLA2G4A and ALOX5AP* gene expressions in leucocytes), or on days 0, 16, 32, 60 days from TAI to measure activities of glutathione peroxidase (GPX), superoxide dismutase (SOD), and concentrations of malondialdehyde (MDA). Total antioxidant capacity (TAC) and P4 concentrations in plasma were monitored after TAI. All gene expression assays were done on blood cell lysates resulting from frozen whole blood. Progesterone concentrations and enzymatic/nonenzymatic responses were determined in plasma.

### Redox balance

The GPX activity in whole blood was measured with a RANSEL kit (RANDOX Laboratories, UK, Cat No. RS 505) using a UV method at 340 nm wavelength^43^. Whole blood was prepared according to the manufacturer’s guidelines (RANDOX Laboratories, UK, Cat No. SD125) and analyzed to determine SOD activity at 505 nm wavelength. Serum TAC concentration was measured using standardized kit (RANDOX Laboratories, UK, Cat No. NX2332) at 600 nm wavelength. An automatic COBAS MIRA PLUS biochemical analyzer (Roche, Switzerland) was used for the measurement of the three indices. Intra- and inter assay coefficients of variation were 4.52 and 4.12% respectively. The detection limits for SOD, GPX, and TAC were 0.1, 40, and 0.05 U/mL, respectively, and the lowest measured concentrations were 0.35, 120, and 0.22 U/mL, respectively. The concentration of malondialdehyde (MDA) was estimated in serum according to the method of Placer et al.^44^.

### Progesterone measurement

Progesterone concentrations measured by enzyme-linked immunosorbent assay (ELISA) kit (DIAPLUS, North York, Canada, Cat No. DP 4926). The intra- and inter assay coefficients of variation were 2.21 and 3.56% respectively.

### Gene expression

#### Total RNA extraction and cDNA synthesis

Total RNA was extracted from whole blood (PMNL) and used for cDNA synthesis using established protocols in our laboratory^45^. Briefly, RNA was isolated using a commercial kit (NUCLEOSIN RNA Blood, Cat No. 40200, Macherey–Nagel GmBH&Co. KG, Büren, Germany).

All RNA samples were quantified by spectrophotometry (#ND-1000, NANODROP Technology Inc., Wilmington, DE, USA) and the purification of RNA with A260/A280 ratio was between 1.9 and 2.3^45^. Complementary DNA (cDNA) was synthesized from 200 ng RNA using a QuantiTect Reverse Transcription kit (QIAGEN, Hilden, Germany, Cat No. 205314). The mixture was incubated at 60 °C for 6 min and kept on ice for 5 min. The reaction was performed in an Eppendorf Mastercycler Gradient using the following temperature program: 25 °C for 5 min, 50 °C for 60 min and 70 °C for 15 min. cDNA was then diluted 1:4 (v:v) with DNase/RNase free water^46^.

#### Real-time PCR

Real-time PCR was performed using a 15 mL reaction volume containing 1 mL single-strand cDNA, 7.5 mL of 1 × SYBR Green master mix (QIAGEN, GmbH, Germany, Cat. No. 204052), 1 mL of each forward and reverse primers and 4.5 mL of distilled H2O in a ROTOR-GENE 6000 Real-Time PCR software (Corbett Research, Sydney, Australia) in accordance with MIQE guidelines^47^ at the following temperature program: 2 min at 50 °C, 10 min at 95 °C, 40 cycles of 15 s at 95 °C, and 1 min at 60 °C. Gene symbols, sequence, and amplicon size of primers are reported in Table 2 (“Supplementary Information”). The internal controls were GOLGA5, SMUG1, and OSBPL2^48,49^. The geometric mean of the internal control genes was used to normalize the expression data. The relative levels of mRNA were analyzed by the 2^−ΔΔCt^ method^50^.

**Table 2:** Features of used primers for qPCR analysis. Sequence and amplicon size of primers for *Bos taurus* used to analyze gene expression.

Symbol	Primers^a^	Primers (5′–3′)	bp^b^
*ALOX5AP*	F	ACAAGGTGGAGCACGAAAGC	100
*ALOX5AP*	R	ACACAGTTCTGGTTGGCAGTGT	
*PLA2G4A*	F	CTCCATGTCAAACCCGATGTC	105
*PLA2G4A*	R	GTCAGGCGCCATAAAAGTACCA	
*IL10*	F	GAAGGACCAACTGCACAGCTT	98
*IL10*	R	AAAACTGGATCATTTCCGACAAG	
*IL1B*	F	ATTCTCTCCAGCCAACCTTCATT	100
*IL1B*	R	TTCTCGTCACTGTAGTAAGCCATCA	
*IL6*	F	CCAGAGAAAACCGAAGCTCTCAT	100
*IL6*	R	CCTTGCTGCTTTCACACTCATC	
*IL8*	F	GACAGCAGAGCTCACAAGCATCT	105
*IL8*	R	AAGCTGCCAAGAGAGCAACAG	
*PTGES*	F	FGGAACGACCCAGATGTGGAA	
*PTGES*	R	GTCCGAGGAAAGAGTAGACAAAGC	
*PTGS2*	F	CGTTTTCTCGTGAAGCCCTATG	102
*PTGS2*	R	CTCCATGGCATCTATGTCTCCAT	
*STAT3*	F	GGTAGCATGTGGGATGGTCTCT	110
*STAT3*	R	GCATCCCTAGAAACTCTGGTCAA	
*TLR2*	F	CCATGTCTGGAGAGGGTGTT	102
*TLR2*	R	GGGGACACAAAACAGCACTT	
*TLR4*	F	GCTGTTTGACCAGTCTGATTGC	100
*TLR4*	R	GGGCTGAAGTAACAACAAGAGGAA	
*TNF*	F	CCAGAGGGAAGAGCAGTCCC	114
*TNF*	R	TCGGCTACAACGTGGGCTAC	

^a^Primer direction (F—forward; R—reverse).

^b^Amplicon size in base pair (bp).

### Statistical analyses

Sample size was set conservatively at 200 cows based on previous designed experiments^2^ n = 100 cows^42^, n = 130 cows) that examined differences in pregnancy statuses and OS in postpartum cows. Present experiment was designed with sufficient power with 200 cows to detect main effects of pregnancy status on gene expression in leukocytes and OS responses in plasma for pregnancy statuses and days of sampling.

All data were analyzed using SAS (Windows; SAS Institute, Cary, NC, USA). All repeated measurement data (SOD, GPX, TAC, and MDA) were analyzed using PROC MIXED of SAS (2001) for repeated measures with the following model:

$$Yijk = \mu + \alpha_{i} + {\text{c}}\left( {\alpha_{j} } \right) + \tau_{k} + \alpha \tau_{ik} + {\text{e}}_{{{\text{ijk}}}}$$

where µ is the population mean, α_i_ is the pregnancy group effect*,* c(*α*_*j*_) is the cows (group) effect, τ_*k*_ is the effect of sampling day after TAI*, (α*τ)_*ik*_ is the interaction effect of pregnancy group and sampling day after TAI, and e_ijk_ is the residual error. Pregnancy groups for the time analyses were non-pregnant (n = 114), pregnant (n = 80) and pregnant-embryo Loss (n = 6).

Milk yield, parity and BCS at AI were included as covariates. Interactions that were not significant (P > 0.10) were excluded from the final model.

Gene expression data are presented as fold changes relative to one of the pregnancy group. These were calculated using the method described by Yuan et al.^51^ whereby fold change were calculated from least square mean difference according to the formula 2^−ΔΔCt^, where ΔCt = Ct_target gene_—geometric mean of Ct _reference genes_, and ΔΔCt = ΔCt_group A_—ΔCt_group B_. Statistical analyses were performed on ΔCt values as described by Livak and Schmittgen^50^. Statistical differences were declared significant at P ≤ 0.05 and tendencies at P ≤ 0.10.

### Approval for animal experiments

The study was conducted in accordance with the guidelines of the Iranian Council of Animal Care (1995) and approved by the ethics committee of Sari Agricultural Sciences and Natural Resources University (protocol #1998).

## Conclusions

Expression of ISG15 mRNA in PBL is elevated at day 16 in pregnant cows compared to nonpregnant cows and pregnant cows that underwent embryo mortality. Forecasting these differential responses may allow for improvements in efficiency of resynchronization programs for second insemination. Chronic higher concentrations of antioxidants in blood, between 0 and 60 days after TAI, occurred in lactating healthy cows that were pregnant at day 16 compared to either non-pregnant or pregnant cows that experienced embryonic losses after day 16. Coupled with the elevations in antioxidants was a concurrent decrease in oxidants associated with peroxidation. Furthermore, expressions of Inflammation associated genes (*STAT3, TLR2, TlR4 IL1β* mRNAs) and Eicosanoid associated genes (ALOX5AP, PLA2G4A, PTGS2 mRNAs) were amplified in peripheral blood leucocytes of day 16 pregnant cows experiencing subsequent pregnancy losses compared to cows maintaining pregnancy to day 60. In contrast PGES mRNA was amplified in pregnant cows. Pregnancy success is associated with the dynamics of OS status.

## Supplementary Information

Supplementary Information.

## Data Availability

None of the data were deposited in an official repository.
