# Supplementary material for: Peripheral leucocyte molecular indicators of inflammation and oxidative stress are altered in dairy cows with embryonic loss
Source: Sci Rep. 2021 Jun 17;11:12771. doi: 10.1038/s41598-021-91535-2 (PMC8211839; doi:10.1038/s41598-021-91535-2)
Supplement: Supplementary file 1 — Supplementary Information. [file 41598_2021_91535_MOESM1_ESM.docx]

**Peripheral leucocyte molecular indicators of inflammation and oxidative stress are altered in dairy cows with embryonic loss**

E. Dirandeh^1^**^*^**, M. A. Sayyar^1^, Z. Ansari-Pirsaraei^1^, H. Deldar^1^ and W.W. Thatcher^2^

| **Additional file 1.** The Function of the genes measured in the polymorphonuclear leukocytes. | |
| --- | --- |
| Inflammation | |
| *STAT3* | Transcription factor that mediates cellular responses to interleukins, and other growth factors |
| *TLR2* | Pathogen recognition and regulation of immune responses |
| *TLR4* | Pathogen recognition and regulation of immune responses |
| *TNF* | Cytokine that regulates inflammatory response |
| Eicosanoids | |
| *ALOX5AP* | Leukotrienes synthesis |
| *PLA2G4A* | Release arachidonic acid from the membrane |
| *PTGS2* | Prostaglandin synthesis |
| Receptors | |
| *IL10* | Cytokine with pleiotropic effects in immunoregulation and inflammation |
| *IL1B* | Cytokine that mediates inflammatory response |
